# Bell’s palsy and obesity, alcohol consumption and smoking: A nested case-control study using a national health screening cohort

**DOI:** 10.1038/s41598-020-61240-7

**Published:** 2020-03-06

**Authors:** So Young Kim, Dong Jun Oh, Bumjung Park, Hyo Geun Choi

**Affiliations:** 1 Department of Otorhinolaryngology-Head & Neck Surgery, CHA Bundang Medical Center, CHA University, Seongnam, Korea; 2 Department of Internal Medicine, Soonchunhyang University College of Medicine, Seoul Hospital, Seoul, Korea; 3 Department of Otorhinolaryngology-Head & Neck Surgery, Hallym University College of Medicine, Anyang, Korea

**Keywords:** Peripheral neuropathies, Risk factors

## Abstract

The aim of this study was to investigate the association of body mass index (BMI), alcohol consumption, and smoking status with the occurrence of Bell’s palsy. The Korean National Health Insurance Service-Health Screening Cohort of a ≥ 40-year-old population from 2000–2003 was used. A total of 5,632 Bell’s palsy participants were matched with 22,528 control participants in terms of age, sex, income, region of residence, and past medical histories of hypertension, diabetes, and dyslipidemia. Bell’s palsy was classified by a history of ≥2 diagnoses with ICD-10 code (G510) and steroid treatment. BMI (kg/m^2^) was classified as <18.5 (underweight), ≥18.5 to <23 (normal), ≥23 to <25 (overweight), ≥25 to <30 (obese I), and ≥30 (obese II). Alcohol consumption was divided into non-drinkers and those who drank 2–3 times a month, 1–2 times a week, and ≥3 times a week. Smoking status was categorized as current smokers, past smokers, and non-smokers. The odds of obesity, alcohol consumption, and smoking with Bell’s palsy were analyzed using logistic regression analysis. BMI showed proportionally positive associations with Bell’s palsy (adjusted OR [95% CI] = 0.61 [0.47–0.79] for underweight, 1.16 [1.08–1.26] for normal, 1.24 [1.15–1.33] for obese I, and 1.61 [1.38–1.88] for obese II, P < 0.001). The odds of alcohol consumption with Bell’s palsy were 0.90 (95% confidence interval [CI] = 0.82–0.99) for 2–3 times a month, 0.77 (95% CI = 0.69–0.85) for 1–2 times a week, and 0.79 (95% CI = 0.71–0.88) for ≥3 times a week compared to nondrinkers (P < 0.001). Smoking did not show a relationship with the occurrence of Bell’s palsy. Obesity was related to the risk of Bell’s palsy in the population over 40 years old. On the other hand, alcohol consumption was negatively associated with the occurrence of Bell’s palsy.

## Introduction

Bell’s palsy is one of the most common types of facial palsy, accounting for approximately 1 in 65 of lifetime prevalence^1^. Approximately 11.5–53.3 per 100,000 people suffer from Bell’s palsy worldwide^2^. In Korea, approximately 0.057% of the population is diagnosed annually with Bell’s palsy^3^. The incidence of Bell’s palsy is not different between the sexes and covers all age populations, with the peak incidence observed in the population aged 15–50 years old^4,5^. Because it is diagnosed by exclusion of other organic causes, the exact pathophysiologic causes of Bell’s palsy remain unknown. Instead, several plausible causes have been proposed, including viral infection, autoimmune inflammatory disease, microangiopathic ischemia, and inflammatory neuritis^6^. Thus, a number of epidemiologic studies have investigated possible predisposing factors of Bell’s palsy, namely, cardiovascular and metabolic factors. Hypertension and diabetes were previously reported to be related to the occurrence of Bell’s palsy^4,7^. However, most previous studies were based on a small number of cases and lacked control groups.

Lifestyle factors including body mass index (BMI), alcohol consumption, and smoking status can influence the risk of chronic disorders such as hypertension and diabetes. For instance, BMI is predictive for the risk of hypertension after adjusting for other variables such as age, ethnicity, marital status, education level, smoking status, alcohol consumption, physical activity, and diabetes^8^. Therefore, these other lifestyle factors could influence the risk of Bell’s palsy via increased cardiovascular and metabolic comorbidities. A previous study reported that obese patients exhibit a poor recovery from Bell’s palsy^9^. However, no prior studies included a normal control group to evaluate the risk of Bell’s palsy according to BMI. In addition, to the best of our knowledge, there has been no research into the association of lifestyle factors of alcohol consumption and smoking with Bell’s palsy. When PubMed and EMBASE databases were searched for articles with the terms “facial palsy [All Fields]” AND “alcohol”[All Fields], no articles were retrieved.

We hypothesized that obesity, alcohol consumption, and smoking could elevate the risk of Bell’s palsy. To test this hypothesis, the matched control groups for age, sex, income, region of residence, and past medical histories of hypertension, diabetes, and dyslipidemia were compared with Bell’s palsy group for the odds of obesity, alcohol consumption, and smoking.

## Results

The BMI distributions were different between the Bell’s palsy group and the control group (P < 0.001, Table 1), as 9.8% (550/4,466) of Bell’s palsy and 11.0% (2,478/17,202) of control participants drink alcohol ≥3 times a week (P < 0.001). The rates of smoking were comparable between groups (P = 0.862). Age, sex, income, region of residence, and past medical histories of hypertension, diabetes, and dyslipidemia were matched between the Bell’s palsy and control groups (all P = 1.00).

**Table 1:** General characteristics of the participants.

Characteristics	Total participants		
	Bell’s palsy (n, %)	Control (n, %)	P-value
Age (years old)			1.000
40–44	132 (2.3)	528 (2.3)	
45–49	644 (11.4)	2,576 (11.4)	
50–54	1,041 (18.5)	4,164 (18.5)	
55–59	989 (17.6)	3,956 (17.6)	
60–64	899 (16.0)	3,596 (16.0)	
65–69	820 (14.6)	3,280 (14.6)	
70–74	607 (10.8)	2,428 (10.8)	
75–79	339 (6.0)	1,356 (6.0)	
80–84	137 (2.4)	548 (2.4)	
85+	24 (0.4)	96 (0.4)	
Sex			1.000
Male	2,807 (49.8)	11,228 (49.8)	
Female	2,825 (50.2)	11,300 (50.2)	
Income			1.000
1 (lowest)	860 (15.3)	3,440 (15.3)	
2	696 (12.4)	2,784 (12.4)	
3	858 (15.2)	3,432 (15.2)	
4	1,291 (22.9)	5,164 (22.9)	
5 (highest)	1,927 (34.2)	7,708 (34.2)	
Region of residence			1.000
Urban	2,441 (43.3)	9,764 (43.3)	
Rural	3,191 (56.7)	12,764 (56.7)	
Hypertension			1.000
Yes	2,172 (38.6)	8,688 (38.6)	
No	3,460 (61.4)	13,840 (61.4)	
Diabetes			1.000
Yes	1,105 (19.6)	4,420 (19.6)	
No	4,527 (80.4)	18,108 (80.4)	
Dyslipidemia			1.000
Yes	748 (13.3)	2,992 (13.3)	
No	4,884 (86.7)	19,536 (86.7)	
BMI (kg/m^2^)			<0.001*
Underweight	70 (1.2)	506 (2.2)	
Normal	1,677 (29.8)	7,527 (33.4)	
Overweight	1,574 (27.9)	6,142 (27.3)	
Obese I	2,047 (36.3)	7,592 (33.7)	
Obese II	264 (4.7)	761 (3.4)	
Smoking			0.862
Nonsmoker	4,078 (72.4)	16,253 (72.1)	
Past smoker	635 (11.3)	2,531 (11.2)	
Current smoker	919 (16.3)	3,744 (16.6)	
Drinking alcohol			<0.001*
Nondrinker	3,747 (66.5)	14,292 (63.4)	
2–3 times a month	719 (12.8)	2,910 (12.9)	
1–2 times a week	616 (10.9)	2,848 (12.6)	
≥3 times a week	550 (9.8)	2,478 (11.0)	

BMI: body mass index, kg/m^2^.

Underweight: <18.5, normal: ≥18.5 to <23, overweight: ≥23 to <25, obese I: ≥ 25 to <30, obese II: ≥ 30.

*Chi-square test; significant at P < 0.05.

Compared with normal BMI, underweight subjects showed decreased odds for Bell’s palsy (adjusted OR = 0.61, 95% CI = 0.47–0.79, P < 0.001; Table 2). Conversely, higher BMI groups demonstrated higher odds for Bell’s palsy, with positive linear associations observed (adjusted OR [95% CI] = 1.16 [1.08–1.26] for overweight, 1.24 [1.15–1.33] for obese I, and 1.61 [1.38–1.88] for obese II, P < 0.001; Table 2). Smoking did not show differences for the odds for Bell’s palsy (P = 0.594; Table 2). Alcohol consumption was associated with decreased odds for Bell’s palsy (adjusted OR [95% CI] for 2–3 times a month = 0.90 [0.82–0.99], adjusted OR [95% CI] for 1–2 times a week = 0.77 [0.69–0.85], adjusted OR [95% CI] for ≥3 times a week = 0.79 [0.71–0.88], P < 0.001; Table 2).

**Table 2:** Crude and adjusted odds ratios (95% confidence interval) of smoking, drinking alcohol, and obesity for Bell’s palsy.

Characteristics	Crude model		Adjusted model	
	Crude^†^	P-value	Adjusted^†,‡^	P-value
Smoking		0.830		0.594
Current smoker	0.97 (0.89–1.07)	0.545	1.05 (0.95–1.15)	0.321
Past smoker	0.99 (0.90–1.10)	0.912	1.03 (0.93–1.15)	0.573
Nonsmoker	1.00		1.00	
Alcohol		<0.001*		<0.001*
≥3 times a week	0.80 (0.72–0.89)	<0.001*	0.79 (0.71–0.88)	<0.001*
1–2 times a week	0.78 (0.71–0.86)	<0.001*	0.77 (0.69–0.85)	<0.001*
2–3 times a month	0.91 (0.82–1.00)	0.039*	0.90 (0.82–0.99)	0.025*
Nondrinker	1.00		1.00	
BMI (kg/m^2^)		<0.001*		<0.001*
Underweight	0.61 (0.47–0.79)	<0.001*	0.61 (0.47–0.79)	<0.001*
Normal	1.00		1.00	
Overweight	1.16 (1.08–1.26)	<0.001*	1.16 (1.08–1.26)	<0.001*
Obese I	1.23 (1.15–1.33)	<0.001*	1.24 (1.15–1.33)	<0.001*
Obese II	1.60 (1.38–1.87)	<0.001*	1.61 (1.38–1.88)	<0.001*

BMI: body mass index, kg/m^2^.

Underweight: <18.5, normal: ≥18.5 to <23, overweight: ≥23 to <25, obese I: ≥ 25 to <30, obese II: ≥ 30.

*Conditional logistic regression analysis; significant at P < 0.05.

^†^Models stratified by age, sex, income, region of residence, hypertension, diabetes, and dyslipidemia.

^‡^Adjusted model of obesity, smoking state (current smokers and past smokers compared to nonsmokers), and frequency of drinking alcohol (≥3 times a week, 1–2 times a week, and 2–3 times a month compared to nondrinkers).

According to age subgroups, higher BMI showed positively increasing odds for Bell’s palsy in both age groups (adjusted OR [95% CI] = 0.67 [0.44–1.01] for underweight, 1.24 [1.11–1.38] for overweight, 1.24 [1.11–1.37] for obese I, and 1.66 [1.33–2.05] for obese II, P < 0.001 for the <60-year-old group and adjusted OR [95% CI] = 0.57 [0.41–0.79] for underweight, 1.09 [0.98–1.22] for overweight, 1.24 [1.12–1.38] for obese I, and 1.56 [1.26–1.94] for obese II, P < 0.001 for the ≥60-year-old group; Table 3). Both the <60-year-old and ≥ 60-year-old groups demonstrated lower odds for Bell’s palsy with alcohol consumption (adjusted OR [95% CI] for 2–3 times a month = 0.94 [0.83–1.06], adjusted OR [95% CI] for 1–2 times a week = 0.78 [0.68–0.89], adjusted OR [95% CI] for ≥3 times a week = 0.82 [0.71–0.96], P = 0.001 for the <60-year-old group and adjusted OR [95% CI] for 2–3 times a month = 0.84 [0.72–0.98], adjusted OR [95% CI] for 1–2 times a week = 0.76 [0.64–0.90], adjusted OR [95% CI] for ≥3 times a week = 0.76 [0.65–0.89], P < 0.001 for the ≥60-year-old group; Table 3).

**Table 3:** Crude and adjusted odds ratios (95% confidence interval) of smoking, drinking alcohol, and obesity for Bell’s palsy in each stratified group according age and sex.

Characteristics	Crude model		Adjusted model	
	Crude	P-value	Adjusted^†^	P-value
**<60 years old (n** = **14,030)**				
Smoking		0.460		0.608
Current smoker	0.95 (0.84–1.07)	0.393	1.02 (0.89–1.15)	0.811
Past smoker	1.04 (0.90–1.20)	0.590	1.08 (0.93–1.25)	0.325
Nonsmoker	1.00		1.00	
Alcohol		0.002*		0.001*
≥3 times a week	0.84 (0.72–0.97)	0.020*	0.82 (0.71–0.96)	0.012*
1–2 times a week	0.79 (0.69–0.90)	<0.001*	0.78 (0.68–0.89)	<0.001*
2–3 times a month	0.95 (0.84–1.07)	0.378	0.94 (0.83–1.06)	0.298
Nondrinker	1.00		1.00	
BMI (kg/m^2^)		<0.001*		<0.001*
Underweight	0.67 (0.44–1.01)	0.055	0.67 (0.44–1.01)	0.056
Normal	1.00		1.00	
Overweight	1.24 (1.11–1.38)	<0.001*	1.24 (1.11–1.38)	<0.001*
Obese I	1.23 (1.11–1.37)	<0.001*	1.24 (1.11–1.37)	<0.001*
Obese II	1.65 (1.33–2.04)	<0.001*	1.66 (1.33–2.05)	<0.001*
**≥60-year-old (n** = **14,130)**				
Smoking		0.707		0.352
Current smoker	1.01 (0.88–1.16)	0.862	1.10 (0.95–1.27)	0.197
Past smoker	0.95 (0.81–1.10)	0.463	0.98 (0.84–1.14)	0.804
Nonsmoker	1.00		1.00	
Alcohol		<0.001*		<0.001*
≥3 times a week	0.77 (0.66–0.89)	0.001*	0.76 (0.65–0.89)	0.001*
1–2 times a week	0.78 (0.66–0.92)	0.003*	0.76 (0.64–0.90)	0.002*
2–3 times a month	0.85 (0.73–0.99)	0.032*	0.84 (0.72–0.98)	0.025*
Nondrinker	1.00		1.00	
BMI (kg/m^2^)		<0.001*		<0.001*
Underweight	0.58 (0.42–0.80)	0.001*	0.57 (0.41–0.79)	0.001*
Normal	1.00		1.00	
Overweight	1.09 (0.97–1.21)	0.136	1.09 (0.98–1.22)	0.128
Obese I	1.24 (1.11–1.37)	<0.001*	1.24 (1.12–1.38)	<0.001*
Obese II	1.56 (1.26–1.93)	<0.001*	1.56 (1.26–1.94)	<0.001*
**Men (n** = **14,035)**				
Smoking		0.816		0.544
Current smoker	0.97 (0.88–1.07)	0.577	1.05 (0.95–1.16)	0.319
Past smoker	1.01 (0.90–1.12)	0.924	1.05 (0.94–1.17)	0.419
Nonsmoker	1.00		1.00	
Alcohol		<0.001*		<0.001*
≥3 times a week	0.79 (0.71–0.89)	<0.001*	0.78 (0.69–0.88)	<0.001*
1–2 times a week	0.77 (0.68–0.86)	<0.001*	0.75 (0.67–0.85)	<0.001*
2–3 times a month	0.90 (0.80–1.02)	0.091	0.89 (0.79–1.00)	0.058
Nondrinker	1.00		1.00	
BMI (kg/m^2^)		<0.001*		<0.001*
Underweight	0.62 (0.44–0.89)	0.008*	0.62 (0.43–0.88)	0.007*
Normal	1.00		1.00	
Overweight	1.20 (1.08–1.34)	0.001*	1.20 (1.08–1.34)	0.001*
Obese I	1.23 (1.11–1.37)	<0.001*	1.23 (1.11–1.37)	<0.001*
Obese II	1.99 (1.57–2.51)	<0.001*	2.00 (1.58–2.53)	<0.001*
**Women (n** = **14,125)**				
Smoking		0.594		0.565
Current smoker	1.00 (0.75–1.33)	0.984	1.07 (0.80–1.44)	0.629
Past smoker	0.76 (0.45–1.28)	0.307	0.78 (0.46–1.31)	0.347
Nonsmoker	1.00		1.00	
Alcohol		0.247		0.206
≥3 times a week	0.87 (0.62–1.20)	0.382	0.86 (0.62–1.19)	0.356
1–2 times a week	0.84 (0.66–1.06)	0.145	0.83 (0.65–1.05)	0.115
2–3 times a month	0.90 (0.77–1.06)	0.197	0.90 (0.77–1.05)	0.186
Nondrinker	1.00		1.00	
BMI (kg/m^2^)		<0.001*		<0.001*
Underweight	0.60 (0.42–0.88)	0.008*	0.60 (0.42–0.87)	0.007*
Normal	1.00		1.00	
Overweight	1.13 (1.01–1.25)	0.034*	1.13 (1.01–1.26)	0.033*
Obese I	1.24 (1.12–1.37)	<0.001*	1.24 (1.12–1.38)	<0.001*
Obese II	1.39 (1.14–1.70)	0.001*	1.39 (1.14–1.70)	0.001*

BMI: body mass index, kg/m^2^.

Underweight: <18.5, normal: ≥18.5 to <23, overweight: ≥23 to <25, obese I: ≥25 to <30, obese II: ≥30.

*Conditional logistic regression analysis; significant at P < 0.05.

^†^Models stratified by age, sex, income, region of residence, hypertension, diabetes, and dyslipidemia.

^‡^Adjusted model of obesity, smoking state (current smokers and past smokers compared to nonsmokers), and frequency of drinking alcohol (≥3 times a week, 1–2 times a week, and 2–3 times a month compared to nondrinkers).

According to sex, both men and women demonstrated proportionally higher odds for Bell’s palsy with higher BMI (adjusted OR [95% CI] = 0.62 [0.43–0.88] for underweight, 1.20 [1.08–1.34] for overweight, 1.23 [1.11–1.37] for obese I, and 2.00 [1.58–2.53] for obese II, P < 0.001 for men and adjusted OR [95% CI] = 0.60 [0.42–0.87] for underweight, 1.13 [1.01–1.26] for overweight, 1.24 [1.12–1.38] for obese I, and 1.39 [1.14–1.70] for obese II, P < 0.001 for women; Table 3). For alcohol consumption, men but not women showed lower odds for Bell’s palsy with alcohol consumption (adjusted OR [95% CI] for 2–3 times a month = 0.89 [0.79–1.00], adjusted OR [95% CI] for 1–2 times a week = 0.75 [0.67–0.85], adjusted OR [95% CI] for ≥3 times a week = 0.78 [0.69–0.88], P < 0.001; Table 3).

## Discussion

Higher BMI was related to higher odds for Bell’s palsy in the present study. Alcohol consumption ≥1 time per week was associated with lower odds for Bell’s palsy. These results were consistent in subgroups according to age and sex. To the best of our knowledge, this study is the first to investigate the relationship between alcohol consumption and Bell’s palsy.

The adverse cardiovascular and metabolic effects of obesity might increase the risk of Bell’s palsy. Obesity is associated with numerous cardiovascular and metabolic diseases, including hypertension, diabetes, and metabolic syndrome. Because these cardiovascular and metabolic disorders were suggested as risk factors for Bell’s palsy, obesity could indirectly influence the risk of Bell’s palsy due to these cardiovascular and metabolic comorbidities. Several previous studies have reported that the risk of Bell’s palsy is associated with hypertension, diabetes, and metabolic syndromes^4,7,10^. Hypertension was related to the onset of Bell’s palsy in ≥40-year-old patients^4^. Although the pathophysiologic mechanisms underlying the relationship between hypertension and Bell’s palsy remain elusive and controversial, fluid retention or edema of the vasa nervorum and increased susceptibility to viral infection due to higher cortisol levels could explain the elevated risk of Bell’s palsy in hypertension patients, as in pregnancy^11^. Diabetes has also been proposed as a risk factor for Bell’s palsy^7,10^. A retrospective study reported that as many as 31.5% and 29% of Bell’s palsy patients had diabetes mellitus and hypertension, respectively^10^. A prospective study demonstrated that abnormally elevated serum glycosylated hemoglobin (HbA1c) levels were related to the severity grade of Bell’s palsy (OR = 4.7, 95% CI = 1.4–15.2, P = 0.008)^7^. The elevated levels of inflammatory cells and inflammatory cytokines in diabetic patients could induce diabetic peripheral facial neuropathy^10^. No study has shown a relationship between BMI and the onset of Bell’s palsy. However, retrospective studies demonstrated that subjects with obesity demonstrated a lower recovery rate of Bell’s palsy than normal subjects^9,12^. In addition, patients with metabolic syndrome showed a lower recovery rate from Bell’s palsy than control subjects^13^.

Contrary to our expectation, alcohol consumption was negatively associated with the risk of Bell’s palsy in this study. It is possible that the biased control group may have affected the relationship between alcohol and Bell’s palsy. To minimize the potential selection bias of the control group, selection of the control group was repeated with a novel random number in this study. Results consistent with the low odds of Bell’s palsy with alcohol consumption were still observed when the new control group was used. The neuroprotective and cardioprotective effects of mild to moderate alcohol consumption may be linked to a reduced risk of Bell’s palsy^14^. A meta-analysis showed that multiple cardiovascular outcomes of coronary heart disease and stroke were decreased in moderate alcohol consumption groups^15^. Moderate alcohol consumption has been suggested to induce anti-inflammatory responses related to adenosine receptors, protein kinase C, nitric oxide synthase, and heat shock proteins, thereby resulting in cardioprotective effects^14,16^. In addition, alcohol exerted direct neuroprotective effects against ischemic, endotoxin, beta-amyloid, and neuroinflammatory proteins, probably by activating reactive oxygen species, a number of protein kinases, and heat shock proteins^14,17^. There has been some evidence supporting the neuroprotective roles of alcohol consumption in epidemiologic studies. For example, cognitive function showed a proportionally positive association with the amount of alcohol consumption in a cross-sectional study^18^. In summary, these cardiovascular and neural effects of alcohol could have led to the protective effects against Bell’s palsy observed in this study. Because only 21% of participants consumed alcohol ≥1 time per week in our cohort, the relatively moderate frequency of alcohol consumption of ≥1 time per week was used as the reference criterion for alcohol consumption. Thus, the negative effects of higher-frequency alcohol consumption could be masked due to the low threshold for alcohol consumption used in this study.

In the subgroup analysis according to age and sex, obesity consistently increased the odds of Bell’s palsy. On the other hand, alcohol consumption decreased the odds of Bell’s palsy in men. However, no relationship between alcohol consumption and Bell’s palsy was found in women. The small number of participants with alcohol consumption ≥1 time per week may have weakened the statistical power in the women subgroup (Supplementary Table S1). Women are presumed to be more susceptible to the effects of alcohol consumption, such as alcohol-related liver diseases, cardiomyopathy, and breast cancer^19^. Thus, further studies that include a sufficient number of participants who consume alcohol are warranted.

A large, nationwide, population-based cohort was used in the present study. The large number of participants enabled the selection of a sufficient number of control participants matched for age, sex, income, region of residence, and past medical histories. Because this study was based on health claim codes, matching of socioeconomic factors was crucial because these factors determine medical accessibility. In addition, the fidelity of the sample cohort was verified in a previous study^20^. No missing participants are expected because the national health insurance system of Korea legally registers and manages all medical records of Korean citizens. Moreover, the relationship between lifestyle factors, including alcohol consumption and smoking, and the onset of Bell’s palsy were evaluated using the most recent health check-up data before the index date (onset of Bell’s palsy) in this study. The participants who did not undergo a health check-up before the index date were excluded.

Nevertheless, a few limitations should be considered when interpreting the present results. On the one hand, because the diagnosis of Bell’s palsy was based on the ICD-10 codes and treatment histories, subclinical or spontaneous recovery cases were excluded from this study. On the other hand, acute facial palsy other than Bell’s palsy could be included in this study due to incorrect ICD-10 coding. In addition, the severity and treatment responses of Bell’s palsy could not be assessed. Regarding lifestyle factors, recall bias is possible for alcohol consumption and smoking because they were surveyed based on a questionnaire. In addition, the total amount of alcohol consumption and smoking could not be calculated because a categorical questionnaire was used. Finally, although this study considered the lifestyle factors of obesity, smoking, and alcohol consumption, possible confounders such as caffeine consumption still existed. Because caffeine consumption might be associated with alcohol reward or alcohol-seeking behavior, alcohol consumption could be correlated with caffeine consumption^21^. Thus, the possible protective effect of caffeine consumption on the risk of Bell’s palsy could mediate the negative association between Bell’s palsy and alcohol consumption in this study.

In conclusion, obesity was associated with higher odds of Bell’s palsy in the ≥40-year-old population. Alcohol consumption ≥1 time per week was related to lower odds of Bell’s palsy than alcohol consumption <1 time per week.

## Materials and Methods

### Study population and data collection

The Ethics Committee of Hallym University (2017-I102) approved the use of these data. The study was exempted from the need for written informed consent by the Institutional Review Board. All analyses adhered to the guidelines and regulations of the Ethics Committee of Hallym University.

### Participant selection

**Figure 1:**
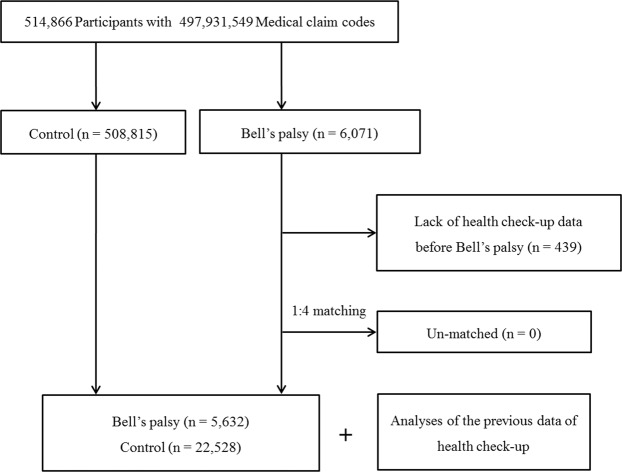
A schematic illustration of the participant selection process used in the present study. Of a total of 514,866 possible participants, 5,632 Bell’s palsy patients were matched with 22,528 control participants for age group, sex, income group, region of residence, and past medical histories.

The Korean National Health Insurance Service-Health Screening Cohort (NHIS-HEALS) 2002–2013 was used^20,22^. This was a nested case-control study. The participants with Bell’s palsy were included in this study (n = 6,071). The control subjects who were never diagnosed with Bell’s palsy were randomly selected from the entire population (n = 508,815) and 1:4 matched with the Bell’s palsy participants. The matching variables were age group, sex, income group, region of residence, hypertension, diabetes, and dyslipidemia. The index date was defined as the date of diagnosis with Bell’s palsy. The index date of control participants was defined as the identical date of their matched Bell’s palsy participants. Bell’s palsy participants without health check-up data before index data were excluded (n = 439). Resultantly, 5,632 Bell’s palsy participants and 22,528 control participants were enrolled. The latest health check-up data before the index date were analyzed for the Bell’s palsy and control groups (Fig. 1).

### Variables

#### Independent variable

Tobacco smoking was defined by current smoking state, duration of smoking, and amount of cigarette smoking (Supplementary Table S2). Smoking status was categorized into 3 groups: current smoker, past smoker, and nonsmoker.

Drinking alcohol was categorized as frequency and the amount of alcohol consumption (Supplementary Table S2). The frequency of alcohol consumption was analyzed in subsequent analyses. BMI (body mass index, kg/m^2^) was categorized as <18.5 (underweight), ≥18.5 to <23 (normal), ≥23 to <25 (overweight), ≥25 to <30 (obese I), and ≥30 (obese II) following WPRO 2000^23^.

#### Covariate analysis

Age groups were classified using 5-year age intervals. The income groups were divided into 5 classes (class 1 [lowest income]-5 [highest income]). The region of residence was grouped into urban and rural areas.

The participants’ prior medical histories were evaluated using ICD-10 codes. Hypertension (I10 and I15), diabetes (E10-E14), and dyslipidemia (E78) were classified based on the number of treatments (≥2) in their medical history.

#### Dependent variable

Bell’s palsy was defined using ICD-10 code (G510). We only included participants who were treated for Bell’s palsy ≥1 time with steroid treatment.

### Statistical analyses

Chi-square tests were used to compare the general characteristics between Bell’s palsy and control groups.

To analyze the odds ratio (OR) of smoking, drinking alcohol, and obesity with Bell’s palsy, conditional logistic regression analysis was used. Models were stratified by age, sex, income, region of residence, hypertension, diabetes, and dyslipidemia. In this analysis, a crude (simple), adjusted model (adjusted model for obesity, smoking state, and frequency of drinking alcohol) was used. The 95% confidence interval (CI) was calculated.

For the subgroup analyses, we divided the participants according to age and sex (<60 years old and ≥60 years old, men and women). The age threshold for age groups was determined as the median value of the total number of participants.

Two-tailed analyses were conducted, and P values less than 0.05 were considered to indicate significance. The results were statistically analyzed using SPSS v. 22.0 (IBM, Armonk, NY, USA).

## Supplementary information

supplement tables.
