# Supplementary material for: Bell’s palsy and obesity, alcohol consumption and smoking: A nested case-control study using a national health screening cohort
Source: Sci Rep. 2020 Mar 6;10:4248. doi: 10.1038/s41598-020-61240-7 (PMC7060281; doi:10.1038/s41598-020-61240-7)
Supplement: Supplementary file 1 — supplement tables. [file 41598_2020_61240_MOESM1_ESM.docx]

**Bell's Palsy and obesity, alcohol consumption and smoking: A nested case-control study using a national health screening cohort**

So Young Kim, MD^1^, Dong Jun Oh, MD^2^, Bumjung Park, MD^3^, Hyo Geun Choi, MD^3*^

^1^Department of Otorhinolaryngology-Head & Neck Surgery, CHA Bundang Medical Center, CHA University, Seongnam, Korea

^2^Department of Internal medicine, Asan Medical Center, University of Ulsan College of Medicine, Seoul

^3^Department of Otorhinolaryngology-Head & Neck Surgery, Hallym University College of Medicine, Anyang, Korea

**Running title:** Obesity, smoking, drinking alcohol, and Bell’s palsy

**Key words:** Bell Palsy; Obesity; Alcohols; Smoking; Risk Factors

*Correspondence: Hyo Geun Choi

**Supplementary table S1** The rates of smoking, alcohol drinking, and obesity according to age group and sex.

| Characteristics | | Age group | | | Sex | | |
| --- | --- | --- | --- | --- | --- | --- | --- |
|  | | <60 years old | ≥60 years old | P-value | Men | Women | P-value |
| Smoking state | |  |  | <0.001* |  |  | <0.001* |
|  | Non-smoker | 9,498 (67.7) | 10,833 (76.7) |  | 6,612 (47.1) | 13,719 (97.1) |  |
|  | Past smoker | 1,620 (11.6) | 1,546 (10.9) |  | 3,060 (21.8) | 106 (0.8) |  |
|  | Current smoker | 2,912 (20.8) | 1,751 (12.4) |  | 4,363 (31.1) | 300 (2.1) |  |
| Drinking alcohol | |  |  | <0.001* |  |  | <0.001* |
|  | Non-drinker | 7,824 (55.8) | 10,215 (72.3) |  | 5,785 (41.2) | 12,254 (86.8) |  |
|  | 2-3 times a month | 2,354 (16.8) | 1,275 (9.0) |  | 2,511 (17.9) | 1,118 (7.9) |  |
|  | 1-2 times a week | 2,287 (16.3) | 1,177 (8.3) |  | 2,960 (21.1) | 504 (3.6) |  |
|  | ≥ 3 times a week | 1,565 (11.2) | 1,463 (10.4) |  | 2,779 (19.8) | 249 (1.8) |  |
| Obesity (BMI, kg/m^2^) | |  |  | 0.141 |  |  | 0.933 |
|  | < 18.5 | 211 (1.5) | 365 (2.6) |  | 303 (2.2) | 273 (1.9) |  |
|  | ≥ 18.5 to < 23 | 4,608 (32.8) | 4,596 (32.5) |  | 4,459 (31.8) | 4,745 (33.6) |  |
|  | ≥ 23 to < 25 | 3,910 (27.9) | 3,806 (26.9) |  | 3,924 (28.0) | 3,792 (26.8) |  |
|  | ≥ 25 to < 30 | 4,791 (34.1) | 4,848 (34.3) |  | 4,962 (35.4) | 4,677 (33.1) |  |
|  | ≥ 30 | 510 (3.6) | 515 (3.6) |  | 387 (2.8) | 638 (4.5) |  |

* Linear by linear association test. Significance at P < 0.05

**Supplementary table S2** Smoking and alcohol consumption

| Characteristics | | | Total participants | | |
| --- | --- | --- | --- | --- | --- |
|  | |  | Bell’s palsy (n, %) | Control (n, %) | P-value |
| Smoking state | | |  |  | 0.619 |
|  | Non-smoker | | 4,078 (72.4) | 16,253 (72.1) |  |
|  | Past smoker | | 635 (11.3) | 2,531 (11.2) |  |
|  | Current smoker | | 919 (16.3) | 3,744 (16.6) |  |
| Duration of smoking (total) | | |  |  | 0.757 |
|  | Non-smoker | | 4,078 (72.4) | 16,253 (72.1) |  |
|  | < 20 years | | 468 (8.3) | 1,907 (8.5) |  |
|  | ≥ 20 years | | 1,086 (19.3) | 4,368 (19.4) |  |
| Current cigarette per a day | | |  |  | 0.986 |
|  | 0 cigarette a day | | 4,713 (83.7) | 18,784 (83.4) |  |
|  | < 20 cigarette a day | | 616 (10.9) | 2,597 (11.5) |  |
|  | ≥ 20 cigarette a day | | 303 (5.4) | 1,147 (5.1) |  |
| Drinking alcohol | | |  |  | <0.001* |
|  | Non-drinker | | 3,747 (66.5) | 14,292 (63.4) |  |
|  | 2-3 times a month | | 719 (12.8) | 2,910 (12.9) |  |
|  | 1-2 times a week | | 616 (10.9) | 2,848 (12.6) |  |
|  | ≥ 3 times a week | | 550 (9.8) | 2,478 (11.0) |  |
| Amount of alcohol in a time | | |  |  | 0.003* |
|  | < Soju 1 bottle | | 4,585 (81.4) | 17,842 (79.2) |  |
|  | ~ Soju 1 bottle | | 752 (13.4) | 3,403 (15.1) |  |
|  | Soju > 1 to < 2 bottle | | 183 (3.3) | 806 (3.6) |  |
|  | ≥ Soju 2 bottle | | 112 (2.0) | 477 (2.1) |  |

* Linear by linear associationtest. Significance at P < 0.05

Their current state of smoking was surveyed as non-smoker, past-smoker, and current smoker.

Duration of smoking was surveyed as non-smoker, < 5 years, 5-9 years, 10-19 years, 20-29 years, ≥ 30 years. It was re-categorized as non-smoker, < 20 year, and ≥ 20 years.

Current cigarette smoking per a day was surveyed as non-smoker, < 10 cigarette, 10-19 cigarette, 20-39 cigarette, and ≥ 40 cigarette. It was re-categorized as 0 cigarette a day, < 20 cigarette a day, and ≥ 20 cigarette a day.

Alcohol habit was surveyed as non-drinker, 2-3 times a month, 1-2 times a week, 3-4 times a week, and ≥ 5 times a week. It was re-categorized as non-drinker, 2-3 times a month, 1-2 times a week, and ≥ 3 times a week.

The amount alcohol intake a time was surveyed as < 1 bottle of Soju, ~ 1 bottle of Soju, > 1 to < 2 bottle of Soju, and ≥ 2 bottle of Soju. Soju is the most common alcohol drink in Korea. Generally, a bottle of Soju contains 17.5% of alcohol with 360 ml. A bottle of Soju is same about 3.5 bottle of bear.
